# New Drugs and Preparations

**Published:** 1895-01-26

**Authors:** 


### NEW DRUGS AND PREPARATIONS.

[We shall be glad to receive, at our Office, 428, Strand, London, W.C., from the manufacturers, specimens of all new preparations and drugs which may be brought out from time to time.]

### USES OF CODEINE.

Pollak (Therap. Monatshefte, Dec., 1893) concludes, regarding codeine (1) That it should be given with caution in doses above one grain. (2) That it acts well as a sedative in diseases of the respiratory tract, in inflammatory conditions of the bowel and peritoneum, and probably in genito-urinary inflammations; but it is useful only for the relief of symptoms. (3) That it is of little value in most painful conditions, and in inflammations of the female generative organs, or as a palliative in the alcohol or opium craving. Dr. Braithwaite (Med. Record, March 16th, 1894) commends codeine highly in nocturnal cough and in vomiting, giving in the former case three-quarters to one grain two hours before going to bed, and in the latter a quarter to half a grain two to four times daily. He has also found it valuable in a form of diarrhœa, which is said to be peculiar to old women, and which consists in the occurrence of one or two liquid stools immediately on rising in the morning. In these cases he gives with good effect a pill containing half to two-thirds grain about four o'clock a.m.

### SPASMOTIN OR SPHACELOTOXIN.

A description of this substance is given in the *Pharmaceutical Journal* of September 29th. It is obtained from ergot, and is a yellow, amorphous powder, insoluble in water and dilute acids, but soluble in alcohol and ether. It is pointed out that great confusion exists regarding the active principles of ergot, partly owing to their instability, but chiefly to the varying conditions of climate, growth, preservation, &c., to which ergot is exposed, and which probably greatly modify its chemical composition. Jacobi

(Therap. Monatsh., 1894, 221) states that the new substance keeps well in powder, but decomposes readily in alkaline solution. In freshly prepared soda solution, or dissolved in alcohol and glycerine, it can be given without provoking local irritation. It was used in Freund's clinic in doses of two-thirds to one and a half grains, and seems to be identical in action with ergot of rye. Its effects are manifest in a few minutes, and reach their maximum in about half an hour later. It apparently closely resembles sphacelinic acid in action, and contracts the small arteries, but it has the advantage of keeping much better.

### PENTAL AS AN ANÆSTHETIC.

Pental or isoamylene $(CH_3)$ C. CH. $CH_3$ is a colourless liquid of low specific gravity, boiling at 38° C, extremely volatile and inflammable, insoluble in water, but mixing freely with alcohol, ether, and chloroform. It has a rather disagreeable odour, but is perfectly unirritating. As an anæsthetic it is best given by means of an inhaler, the open method not being so satisfactory. On inhalation there occurs slight flushing of the face, with quickening of the pulse, but no diminution in its force, respiration becomes deeper and at first more rapid, but ultimately slower. Consciousness is not quite lost with a short inhalation, but pain is abolished. It seems to be safer than chloroform, but dangerous symptoms sometimes supervene and deaths have been reported.

Philip (Archiv. f. Kinderheilkunde xvi., p. 203, 1893) has used pental in over 1,000 cases in children, and recommends it strongly as a general anæsthetic. It is safer than chloroform, and has no unpleasant after-effects. He occasionally observed sudden cyanosis, which he attributes to reflex spasm of the respiratory muscles, and recommends that it should not be used in cases of laryngeal disease, nor where tracheotomy is to be performed.

### COMPOUND TINCTURE OF COAL TAR.

Duhring (Amer. J. Med. Sci., Ap. 1894) states that tincture of coal-tar is best made by digesting one part coal tar with six parts tincture of quillaia for eight days, agitating frequently, and finally filtering. The product is a clear brown-black colour, and on the addition of water forms a yellowish emulsion. It is prescribed as a stimulating wash, diluted with from ten to sixty parts of water, in cases where tar is indicated, as in certain cases of eczema, psoriasis, &c. This preparation is designed to take the place of "liquor carbonis detergens" and similar proprietary tar preparations.

